# Artesunate: attenuating TLR4/MD2 signaling to alleviate cardiac fibrosis

**DOI:** 10.1038/s41392-025-02131-z

**Published:** 2025-01-31

**Authors:** Lars Koch, Konrad Hoeft, Rafael Kramann

**Affiliations:** 1 Department of Medicine 2, RWTH Aachen University, Medical Faculty, Aachen, Germany; 2 Department of Internal Medicine, Nephrology and Transplantation, Erasmus Medical Center, Rotterdam, The Netherlands

**Keywords:** Cardiology, Cardiovascular diseases, Molecular medicine, Drug screening

In a recent study published in *Cell*, Zhang, Thai et al. conducted a high-throughput drug screen on iPSC-derived cardiac fibroblasts, identifying artesunate as a potent antifibrotic compound.^1^ Mechanistically, artesunate interferes with the formation of the TLR4/MD2-signaling complex to suppress profibrotic gene expression, positioning it as a promising candidate for drug repurposing in heart failure.

Fibrosis is the excessive deposition of extracellular matrix (ECM) following injury, disrupting tissue architecture across various organs, and ultimately leading to organ failure. In the heart, ECM deposition initially preserves tissue integrity and prevents cardiac rupture. However, excess ECM accumulation gradually disrupts tissue architecture and drives progressive heart failure. While recent advances have been made in heart failure therapy there is still an unmet clinical need to develop more effective therapies that could be added to the current Fantastic Four regimen. After years of failed research, focusing on cardiomyocytes, cardiac fibrosis becomes an interesting novel therapeutic target in heart failure, both with preserved and reduced ejection fraction.

Pathway-unbiased phenotypic drug screens have recently emerged as a valuable approach for first-in-class drug discovery. However, high-throughput drug screens have been limited by the scarce availability of primary human cardiac fibroblasts, leading most screens to rely on mouse fibroblasts despite significant interspecies differences. Zhang et al. previously developed a protocol to derive cardiac fibroblasts from human induced pluripotent stem cells (iPSCs), effectively providing an unlimited source of human cardiac fibroblasts.^2^

Building on this platform, Zhang, Thai et al. have now conducted a high-throughput screen of ~5000 compounds at seven different concentrations on human iPSC-derived cardiac fibroblasts.^1^ To enable real-time monitoring of myofibroblast activation, they created a reporter cell line by tagging the myofibroblast marker *ACTA2* with the fluorophore *Clover2* using CRISPR-Cas9 gene editing. Cell viability was simultaneously assessed using a Hoechst staining. Twenty compounds that strongly reduced *ACTA2* expression with minimal impact on fibroblast viability were then selected and counter-screened on iPSC-derived cardiomyocytes and endothelial cells, assessing contraction velocity and cell viability. Here, the anti-malaria drug artesunate emerged as a top candidate, showing strong inhibition of ACTA2 expression with no observed toxicity on all tested cell types.

Artesunate is a derivative of artemisinin, a natural compound extracted from *Artemisia annua* (sweet wormwood), which is the first-line treatment for severe malaria. In 2015 its discovery earned the Nobel Prize in Medicine. Its mechanism primarily involves generating reactive oxygen species by binding heme in red blood cells, damaging the parasite’s proteins and membranes. Beyond malaria, artesunate has shown efficacy against various cancers as well as anti-viral and anti-inflammatory effects.^3^ However, its impact on cardiac fibrosis was yet unexplored.

Zhang, Thai et al. studied the effect of artesunate on myofibroblast activation in the iPSC-derived reporter fibroblasts (described above) stimulated with various profibrotic factors, including Angiotensin II, CTGF, IL11, PDGFBB, TGFβ1, TNC, and ET1. Artesunate treatment significantly reduced *ACTA2* expression across all injury stimuli. Following TGFβ treatment, artesunate also inhibited the proliferation, migration, gel contraction and collagen secretion of fibroblasts. In human primary cardiac fibroblasts treatment with artesunate caused a dose-dependent reduction in fibrosis-related proteins, after TGF-β1 and IL-11 stimulation, with diseased myofibroblasts exhibiting a similar response.

Next, the authors evaluated artesunate’s effects in two mouse models of transverse aortic constriction (TAC). In a preventive approach, artesunate was administered intraperitoneally immediately after surgery, while in a therapeutic approach, treatment began four weeks post-surgery. In both models, artesunate-treated mice exhibited reduced collagen deposition, preserved left ventricular ejection fraction, and reduced diastolic dysfunction. A similar protective effect was observed in a myocardial infarction model, where artesunate treatment was initiated three days after injury. Next the authors performed single nucleus RNA sequencing (snRNA-seq) on TAC-injured, artesunate-treated mice and observed that artesunate reversed the injury-associated fibroblast polarization, as shown by pseudotime analysis and a marked reduction in myofibroblast genes such as *Postn* and *Tgfβ1*.

Toll-like receptors (TLRs) are a family of proteins, primarily recognized for their key role in the innate immune system. However, recent studies have highlighted their contribution to fibroblast activation through the recognition of damage-associated molecular patterns (DAMPs), such as the ECM protein Tenascin-C.^4^ Upon ligand binding, TLRs undergo conformational changes to drive proinflammatory and profibrotic intracellular pathways. Artesunate has been reported to inhibit the Toll-like receptor 4 (TLR4) pathway in macrophages after stimulation with lipopolysaccharide (LPS), which explains artesunate’s protective effect in sepsis models.^5^ Myeloid Differentiation Protein 2 (MD2) functions as a co-receptor that, together with TLR4, forms the active signaling receptor complex upon ligand binding.

**Fig. 1:**
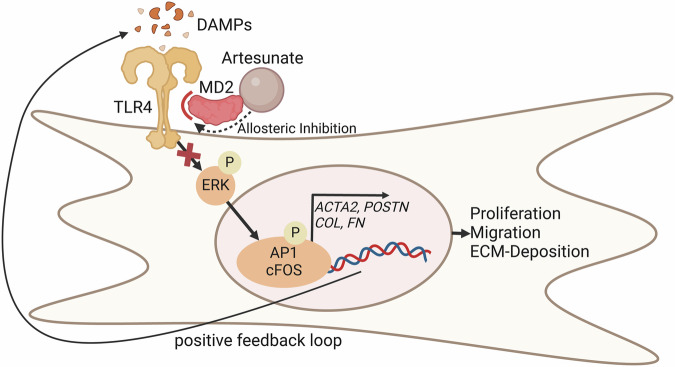
Artesunate binds to the TLR4 coreceptor MD2, allosterically inhibiting the formation of the TLR4/MD2 complex upon DAMP activation. This inhibits ERK and AP1 phosphorylation, ultimately downregulating profibrotic gene expression, responsible for myofibroblast proliferation, migration, and ECM-deposition

Zhang, Thai et al. demonstrated that siRNA knockdown of either MD2 or TLR4 in human cardiac fibroblasts reduced the expression of *ACTA2*, *COL1A1,* and *CTGF*. Using surface plasmon resonance, they confirmed a direct binding of artesunate to MD2. Furthermore, in silico simulations predicted that artesunate stabilizes MD2 residues involved in TLR4 binding, a finding later confirmed by a tryptophan fluorescence assay. Next, the researchers confirmed that artesunate inhibits the MD2-TLR4 interaction using an in-situ proximity ligation assay, as well as co-immunoprecipitation in a HEK-cell line overexpressing both *MD2* and *TLR4*. In line with this observation, Artesunate inhibited ERK phosphorylation, a major downstream signaling pathway of TLR4/MD2, after both LPS and TGFβ1 stimulation (Fig. 1).

To examine the downstream profibrotic effects of the TLR4/MD2 receptor complex, the authors performed ATAC-seq (Assay for Transposase-Accessible Chromatin using sequencing) of human primary cardiac fibroblasts treated with TGFβ1, with and without artesunate treatment. Artesunate inhibited TGFβ1 induced chromatin accessibility at key fibrotic gene loci, including *COL1A1*, *FN1,* or *CTGF*. Unsupervised motif enrichment analysis revealed that the activator protein 1 (AP-1) family, downstream effectors of ERK, was most significantly downregulated. Downregulation of cFOS, a primary AP-1 component, was subsequently validated in primary fibroblasts.

In summary, Zhang, Thai et al. demonstrate that artesunate acts as a potent antifibrotic agent preserving cardiac function in both therapeutic and preventive settings. Mechanistically, artesunate binds to the co-receptor MD2, allosterically inhibiting its interaction with TLR4 and thereby blocking downstream profibrotic signaling pathways, positioning artesunate as an intriguing candidate for drug repurposing in the treatment of cardiac fibrosis and heart failure.
